# Atomistic insights into adhesion characteristics of tungsten on titanium nitride using steered molecular dynamics with machine learning interatomic potential

**DOI:** 10.1038/s41598-023-44265-6

**Published:** 2023-10-10

**Authors:** Eunseog Cho, Won-Joon Son, Eunae Cho, Inkook Jang, Dae Sin Kim, Kyoungmin Min

**Affiliations:** 1 CSE Team, Samsung Electronics, 1 Samsungjeonja-ro, Hwaseong-si, Gyeonggi-do 18448 Republic of Korea; 2 School of Mechanical Engineering, Soongsil University, 369 Sangdo-ro, Dongjak-gu, Seoul, 06978 Republic of Korea

**Keywords:** Atomistic models, Computational methods

## Abstract

As transistor integration accelerates and miniaturization progresses, improving the interfacial adhesion characteristics of complex metal interconnect has become a major issue in ensuring semiconductor device reliability. Therefore, it is becoming increasingly important to interpret the adhesive properties of metal interconnects at the atomic level, predict their adhesive strength and failure mode, and develop computational methods that can be universally applied regardless of interface properties. In this study, we propose a method for theoretically understanding adhesion characteristics through steering molecular dynamics simulations based on machine learning interatomic potentials. We utilized this method to investigate the adhesion characteristics of tungsten deposited on titanium nitride barrier metal (W/TiN) as a representative metal interconnect structure in devices. Pulling tests that pull two materials apart and sliding tests that pull them against each other in a shear direction were implemented to investigate the failure mode and adhesive strength depending on TiN facet orientation. We found that the W/TiN interface showed an adhesive failure where they separate from each other when tested with pulling force on Ti-rich (111) or (001) facets while cohesive failures occurred where W itself was destroyed on N-rich (111) facet. The adhesion strength was defined as the maximum force causing failure during the pulling test for consistent interpretation and the strengths of tungsten were predicted to be strongest when deposited onto N-rich (111) facet while weakest on Ti-rich (111) facet.

## Introduction

As the number of transistors in an integrated circuit chip increases, the structure of the middle and back-end of line (MOL/BEOL) that connects them is becoming more complex, and ensuring the reliability of metal interconnection under allowable current density is becoming increasingly important^1–4^. Device reliability refers to the probability of being able to perform the required function under given conditions for a given period, and it is related to typical wear-out phenomena that occur during long-term usage. In the metal interconnection of MOL/BEOL, the degradation of reliability is induced by an atomic migration by which the typically observed phenomena caused include electromigration (EM)^5,6^, time-dependent dielectric breakdown (TDDB)^7–10^, and stress migration (SM)^11,12^. The EM is caused by the diffusion of atoms in the electron direction resulting in voids when high current flows for a long time at high temperatures on metal interconnection, which often induces breakdown in W and barrier layers like TiN^13,14^. The TDDB is a phenomenon in which breakdown occurs when the dielectric materials that block the connection between metals under high voltage are damaged by an electric field or electron energy, allowing metal atoms to move through and cause a short circuit^10^. The SM is a failure mechanism that occurs in Al interconnect^12,15^ or Sn lead frame^16^, often observed in which hillocks or whiskers are generated to relax stress inside the metal or at the junction between metal and dielectric. Importantly, all typical failure phenomena mentioned above occur at the interface and are strongly related to interfacial adhesion^17–20^. Therefore, it is important to investigate the adhesion characteristics of metal interfaces as it can prevent atomic migration occurring at the interface and improve device reliability.

The various methods to experimentally measure adhesion have been devised, and the representative ones are pull-off test^21,22^ based on continuum mechanics, and peel test^23^, 4-point bending (4PB) test^24,25^, and double cantilever beam (DCB) test^26,27^ based on fracture mechanics. The pull-off test, assuming that there are no cracks in the material where stress is concentrated, involves abruptly delaminating the top materials at once to measure adhesion. This method is highly sensitive to defect distribution in materials, only measures maximum load values, and has limitations when applied to multilayer thin films. On the other hand, for the peel test, the material is bent and then one end is pulled to measure adhesion. Unlike the pull-off test, this method is less affected by defects in material but has limitations when applied to brittle or hard materials that are difficult to bend compared to flexible ones. The 4PB test which is considered the most representative method for measuring adhesion is based on the assumption that it is possible to measure adhesion by creating a notch under the substrate and breaking four points, causing cracks to propagate along the interface. However, this method requires an additional process of forcibly creating the notch under the substrate, and for materials with strong adhesion, it is difficult to conduct the test since they tend to break vertically through the material instead of propagating the crack along the interface. Finally, the DCB test involves creating a notch forcibly to the side of the interface and repeatedly loading and unloading to separate the interface. This method is more suitable for thick films and systems with strong adhesion, but it is known as less realistic than the 4PB test since it can only measure pure tensile stress. Methods such as the peel test, 4PB test, and DCB test based on fracture mechanics involve artificially creating an initial crack and calculating the energy required for crack propagation to predict adhesion; these tests are less sensitive to defect distribution in materials providing intrinsic information about material properties. However, since the phase angle (the ratio of shear to tensile mode) differs among these methods, it is impossible to compare results obtained from different methods^28–30^. Furthermore, as mentioned above, there are also issues with applying different measurement methods depending on material properties such as brittleness or flexibility, and whether they and thin or thick films with weak or strong adhesion.

Therefore, it is necessary to utilize computational methods that can explain adhesion from a consistent perspective and, as the downsizing of devices is accelerated, theoretical interpretations through an atomistic viewpoint become increasingly important. Recently, the steered molecular dynamics (SMD) method has been studied as a computational approach to investigate adhesion in polymeric systems and explain failure characteristics of polymer interfaces within the atomistic framework^31–35^. Conventional MD methods are unable to model such phenomena due to their limited time scale, while material failure occurs over long periods. However, SMD induces delamination or failure mode by applying a constant velocity as an external source and calculates the necessary force and energy at that moment, and as a result, it has been successfully applied in polymer interfacial studies. In particular, the we investigated the adhesion and failure behaviors of polyimide-glass interfaces^31,32^ which are representative materials for flexible display substrates, and demonstrated the effectiveness of the SMD method. However, to our best knowledge, there have been few studies on the adhesion characteristics of metal interfaces using SMD. In the case of heterogeneous metal interfaces, it is necessary to simulate complex surface properties of different materials and various instantaneous structures that may occur during the failure process. This poses a challenging task since it cannot be easily achieved with existing force field methods using predefined simple functional forms.

In this study, we investigated the adhesion characteristics of tungsten deposited on titanium nitride metal (W/TiN), which are representative of the metal interconnect of semiconductor devices^13,14,36–39^. To achieve this goal, we employed a type of machine learning interatomic potential called moment tensor potential (MTP)^40^ and integrated it with SMD. Tungsten is a material that has been used for a long time to fill via hole or the contact in the MOL/BEOL of semiconductor devices due to its low resistivity, high melting point, and excellent gap-filling properties. Moreover, tungsten is also being researched as a candidate material for the metal gate to improve device performance. However, it exhibits poor adhesion, especially, on oxide surfaces^41^ and high reactivity of WF_6_ precursor gas^42,43^ and thus requires TiN as a barrier metal. However, as device miniaturization progresses, there is an increased likelihood of failure occurring at interfaces or tungsten itself. Therefore, it is crucial to theoretically examine the adhesion characteristics of W on various facets of TiN metal.

## Computational methods

### SMD method and MTP potential

To comprehensively understand the adhesion characteristics of interfaces at the atomistic level and evaluate the reliability of metal interconnect, it is necessary to calculate how much force is required when a structure fails, and which part is destructed at that force. However, using the conventional MD simulations, we cannot observe such non-equilibrium phenomena in which materials are destroyed over long periods. As an alternative, the SMD method^44–46^ has been developed, and it allows us to calculate the necessary force and energy while forcibly steering metal interconnect in a desired direction. The theoretical background of the SMD is as follows. Assuming that atoms are connected by virtual springs to dummy atoms, which are known as ghost atoms, during SMD simulation, when the dummy atom moves in a desired direction at a constant velocity, the real atoms connected to it via virtual springs also move. This generates potential energy from which the force applied to real atoms can be calculated. The average work is calculated by Jarzynski’s equality^46^, which relates the equilibrium quantity (i.e., the potential of mean force (PMF)) to the non-equilibrium process, and the PMF value is computed from the following equation.

$$\mathrm{PMF}= -\frac{1}{\upbeta }\mathrm{log}\langle {\mathrm{e}}^{-\mathrm{\beta W}}\rangle$$

where *β* = 1/(*k*_B_*T*) with the Boltzmann constant *k*_B_ and the temperature *T* of the system, *W* is the work done during the SMD, and the bracket indicates the ensemble average of the given quantity.

Meanwhile, in this study, the force field is constructed using moment tensor potential (MTP) which is one of the widely used machine learning interatomic potentials^40,47^. The MTP potential expresses the energy of atomic configurations as a sum of contributions from the local atomic environment of each atom, using the linear combination of the MTP basis function. The necessary parameters for the potential are obtained by fitting a lot of structures and corresponding energy values. Currently, a practical algorithm has been proposed that combines the MTP potential with an active learning method to efficiently update datasets^48^. This approach has been successfully applied to elucidate various materials properties such as finding thermodynamically stable structures of new alloy composition and predicting transport properties of composite materials^49,50^. Here, a potential that can describe various interfaces and failure structures of W and TiN using the MTP method was created. This potential was then combined with SMD simulations to investigate the adhesion characteristics of the W/TiN interconnect.

### Interfacial structure of W on TiN

TiN is a metal with a rocksalt structure in bulk and when it is used as a barrier metal, the major facet exposed on the surface has not been experimentally determined. However, it is generally predicted that several facets will be simultaneously exposed depending on the manufacturing environment. Here, to investigate the adhesion characteristics of TiN depending on its facets, we selected the representative facets of the rocksalt structure, (111) and (001) facets. The (111) facet is a polar one (either Ti or N atoms are exposed), and in a Ti-rich environment, Ti atoms are exclusively exposed on the (111) surface (hereafter referred to as (111)Ti), while in an N-rich, only N atoms are exposed on the surface ((111)N). In contrast, the (001) facet is a nonpolar surface that is not affected by the chemical potential of Ti or N because both Ti and N atoms are exposed on the surface with stoichiometry identical to that of bulk ((001)TiN). Meanwhile, it is known that W deposited on TiN does not exhibit a thermodynamically stable α-W structure but shows an amorphous-like structure in X-ray diffraction^36,37^. Most metals are crystalline in their solid state; therefore, W on TiN does not have a perfect amorphous structure but rather exhibits an imperfectly matched crystal structure compared to α-W, namely, an amorphous-like structure. The methods for creating both the surface structures of TiN and amorphous-like W are described in Supporting Information (SI). To model adhesion, we placed an amorphous W layer on each (111)Ti, (111)N, and (001)TiN structure and optimized the structures to create the W/Ti laminated interconnect. The resulting three different W/TiN structures were labeled as follows: W/(111)Ti for the (111)Ti facet; W/(111)N, for the (111)N facet, and W/(001)TiN for the (001)TiN facet.

### Adhesion test: pulling and sliding methods

**Figure 1:**
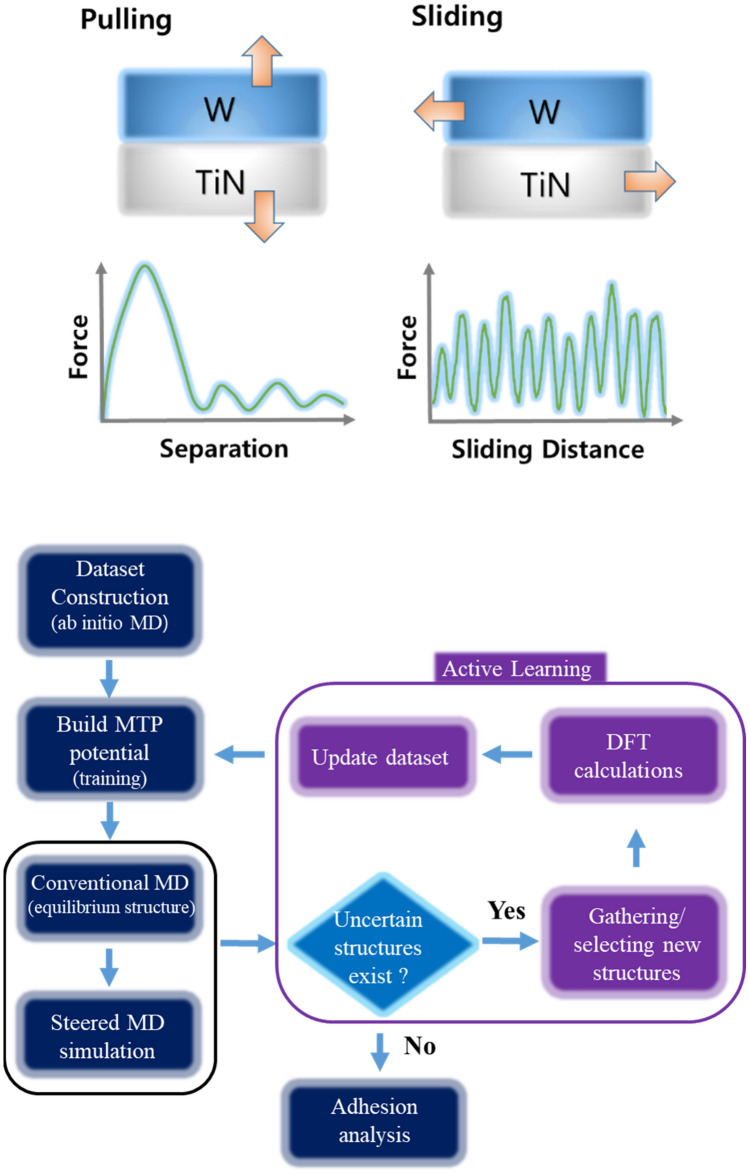
**(a**) Schematic diagram of adhesion testing modes and corresponding force variations for the pulling and sliding tests of W/TiN structure. (**b**) Process for calculating adhesion properties via SMD simulation with generated MTP potential. The black box represents MD simulation steps, and the violet box represents the active learning process that works when uncertain structures beyond the description of trained MTP potential are found during MD simulations.

Adhesion was tested using two methods, pulling and sliding tests, through SMD simulations. Figure 1a shows a diagram of these methods. Pulling involves holding onto both W and TiN separately and pulling them in the direction of separation at the same speed during SMD simulation to record the necessary force when destroying the interface. The typical pattern of changes in required force with respect to the separation distance between two materials is that as they move apart, force is needed because bonding needs to be broken at their interface until it reaches its maximum value. As they move further apart, the interaction between them weakens (the force required for separation decreases), and eventually, when they are completely separated, the force converges to zero. In contrast, sliding involves holding onto both materials and shearing them against each other while recording necessary forces; assuming an infinite length for their interfaces in the modeling means that instead of separating from each other they continue moving by rubbing against one another causing oscillations rather than converging towards zero as typically seen with this method. The sliding distance is defined as the distance between the dummy atoms of W and TiN that move in the direction they are pulled. In this study, we applied pulling method on two different interfacial structures: unit interface structure where minimum size interfaces to eliminate lattice mismatch between W and TiN were considered based on lattice vectors for W/TiN structure (8.971 Å × 15.538 Å for both W/(111)Ti and W/(111)N structures; 8.971 Å × 14.952 Å for W/(001)TiN structure), and 2 × 2 interface structure (4 times larger interfacial area than unit interface). For the sliding test, we used a unit cell size extended three times along the sliding direction, 1 × 3 interface structure. For the unit-interfacial structures, we tested the variation of both spring constant (4.3–43 eV/Å^2^) and velocity (0.01–0.05 Å/ps) and observed the separation of W/TiN and the corresponding force changes over simulation time. When the spring constant is small, during the SMD, the distance between the two materials changes slowly and a sudden separation occurs at a certain time. On the other hand, as the spring constant increases, with increasing time, there is a gradual and consistent change in distance between the two materials. Additionally, in all cases, we found that maximum force required to induce separation remains constant. Furthermore, increasing the spring constant proportionally with an increase in the interfacial size yields more stable simulation results. While lower velocities require longer overall simulation time, they do not significantly alter the overall separation behavior. Based on these findings, SMD simulations employed spring constants set respectively at 40 eV/Å^2^, 160 eV/Å^2^, and 120 eV/Å^2^ for the unit interface, the 2 × 2 interface, and the1 × 3 interface structures, respectively; velocity was fixed at 0.01 Å/ps for all structures.

### Overall simulation workflow

Figure 1b shows the simulation workflow for calculating adhesion properties. First, the W/TiN structure was optimized using the density functional theory (DFT) framework and then ab initio MD simulations were performed to construct an initial dataset with the optimized interfacial structure as its starting point. In this calculation, ab initio MD was performed at 300 K for 10 ps, and trajectory data with energy information was collected every 10 fs resulting in a total of 1000 initial datasets. The obtained dataset was used to train MTP potential which is then utilized in SMD simulations that apply external bias (pulling/sliding) on structures as well as in conventional MD methods to obtain equilibrium structures. Herein, we conducted an SMD calculation for 300 ps after obtaining the equilibrium structure by conventional MD for 500 ps at 300 K. During SMD calculations if uncertain structures (or extrapolated structures) that cannot be predicted by existing MTP potentials are found, they are added to our collection followed by selecting some among them, which is conducted by the Maxvol algorithm^51^ implemented within MLIP package^48,52^. After that, our existing datasets are updated via DFT calculation with selected new structures, and then training is performed with updated datasets again for obtaining new MTP potential. This process is repeated until no more uncertain structures emerge from further iterations, and then simulation results regarding adhesion properties are analyzed. In the early stage of simulation, the MTP potential is primarily developed based on dataset obtained from short ab initio MD results performed for 10 ps. When performing conventional MD for 500 ps to obtain equilibrium structures, new structures frequently emerge that cannot be accurately described with initial MTP potential. Therefore, it is necessary to update the dataset by aggregating these newly generated structures. A more significant challenge arises during SMD simulation, where non-equilibrium structures occur that are difficult to observe in ab initio MD or conventional MD simulations. In order to build an accurate MTP potential, it is necessary to incorporate these non-equilibrium structures into the dataset. The most efficient way to generate such structures is by repeating SMD simulations under the identical conditions as the final production run for adhesion analysis. By iteratively going through this active learning process, updating the dataset and improving existing potential, it becomes possible to build MTP potential that can accurately describe all possible structures that can occur during interfacial delamination in SMD simulation. In this study, we trained MTP potential utilizing a total number of 2973 W/(111)Ti structures, 3084 W/(111)N structures, and 2940 W/(001)TiN structures based on their respective interfacial sizes, and obtained the training error value of 7.3 meV/atom for mean absolute error (MAE) and 13 meV/atom for root mean square error (RMSE). Considering that we initially built the MTP potential using 1000 structures for each facet through ab initio MD method, it can be inferred that through the active learning process, we have added around 2000 structures for each facet. Meanwhile, all DFT calculations including ab initio MD were performed by the generalized gradient approximation (GGA) method^53^, using the Vienna ab initio Simulation Package (VASP)^54,55^, and also D2 correction of Grimme was adopted to treat the van der Waals (vdW) interactions^56^ between W and TiN (calculation details are described in SI).

## Results

**Figure 2:**
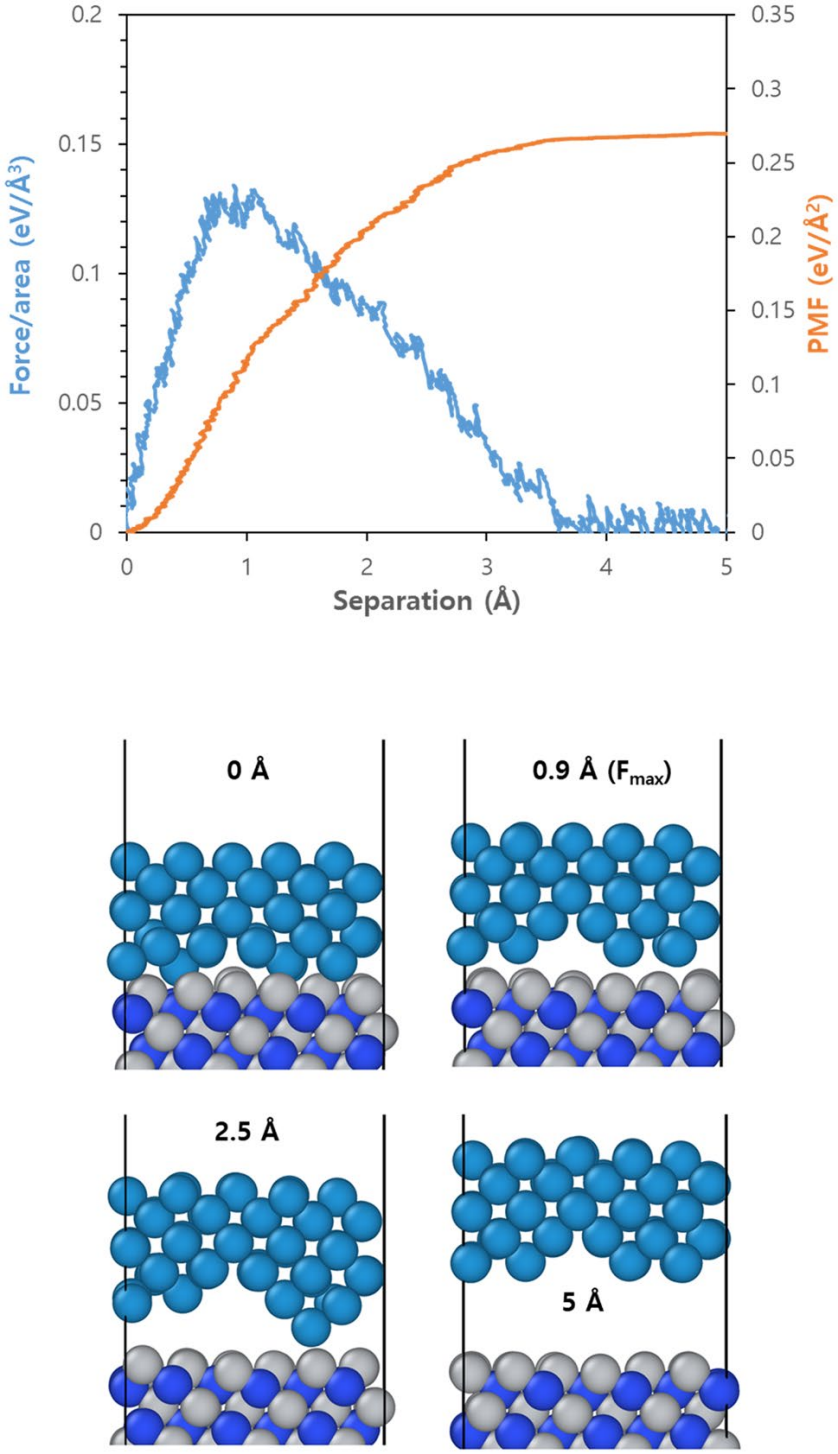
(**a**) Force (blue) and accumulated PMF (red) variations as a function of separation for W/(111)Ti structures with the unit interface when the pulling tests are applied, and (**b**) representative snapshots at four different separation lengths. The number represents the separation distance and the separation of the equilibrium structure is set to 0 Å. F_max_ represents the point at which the force required for separation becomes maximum; W: dark cyan, Ti: gray, and N: blue.

Figure 2a shows the force and PMF changes between W and TiN as the pulling test is performed on the W/(111)Ti structure with the unit interface. The optimized structure obtained by DFT calculations was used as an initial interfacial structure, followed by conventional MD for 500 ps at 300 K to create an equilibrium structure. Then, steered MD was performed to gradually break the bond between W and Ti at the interface until a complete delamination occurred in this heterogeneous interface. At approximately 0.9 Å distance between two metals, force reaches its maximum value of 0.130 eV/Å^3^ required to separate them from each other under the pulling mode. As separation increases further, interaction decreases rapidly resulting in a sharp decrease in force until it becomes almost zero when two materials are separated by about 4 Å distance from each other. Therefore accumulated PMF values also change to variation of forces acting on these two materials during the pulling test since PMF is an average work as explained earlier; it increases along with increasing forces applied on W/TiN structure while converging into constant value when the forces reach zero. The maximum force required for separating these two structures (0.13 eV/Å^3^) can be defined as adhesion force while energy converged (0.27 eV/Å^2^) during that process can be defined as adhesion energy. Figure 2b shows snapshots demonstrating structural changes occurring at specific separations due to performing pulling tests. Note that the 0 Å indicates W/TiN stays in equilibrium structure before SMD simulation. Interestingly W/(111)Ti structure shows ideal adhesive failure where complete separation happens exactly at interfaces without any damage or deformation observed within the bulk region of each metal. Moreover, considering the maximum force attains at only 0.9 Å separation, even short separation for metal interconnects can destroy structures afterward.

**Figure 3:**
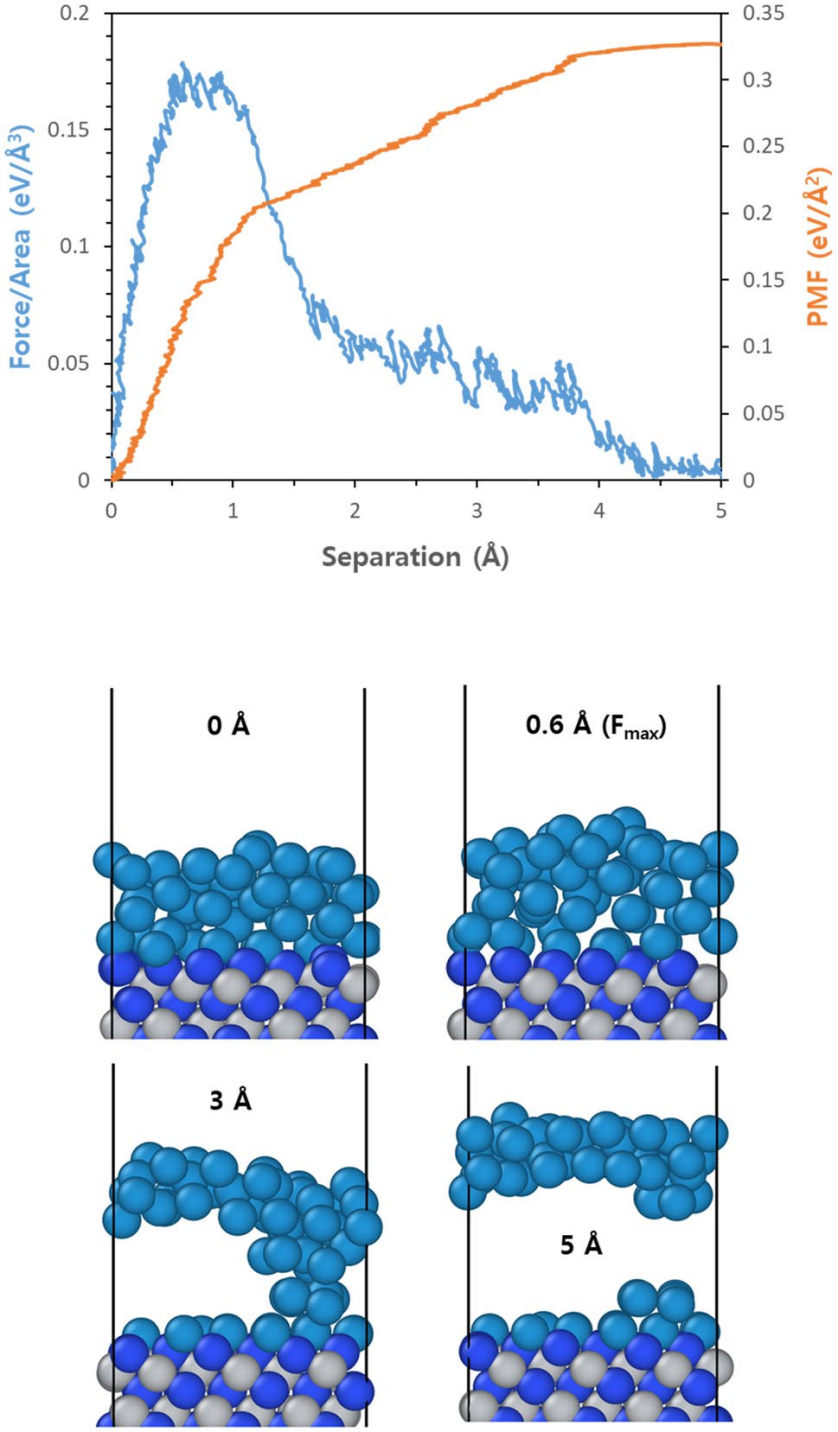
(**a**) Force (blue) and accumulated PMF (red) variations as a function of separation for W/(111)N structures with the unit interface when the pulling tests are applied, and (**b**) representative snapshots at four different separations lengths; W: dark cyan, Ti: gray, and N: blue.

Figure 3 shows the adhesion properties and structural changes for the W/(111)N structure. The structure reaches its maximum force of 0.164 eV/Å^3^ at a separation distance of 0.6 Å when the pulling mode is activated, and unlike the W/(111)Ti structure, it shows slightly more complex force variations without immediately converging to zero force. The reason for this behavior can be seen from the snapshot in Fig. 3b, where cohesive failure occurs with tungsten being destroyed instead of separating at interfaces between the W and (111)N facets. Specifically, at maximum force, the interior of the W shows to create a void rather than the interface, and finally even at further separation, a considerable portion of W atoms still remain on the interface. This is because the adhesion at interfaces is stronger in the case of W/(111)N structures than W/(111)Ti, and thus they exhibit phenomena where W itself is destroyed.

**Figure 4:**
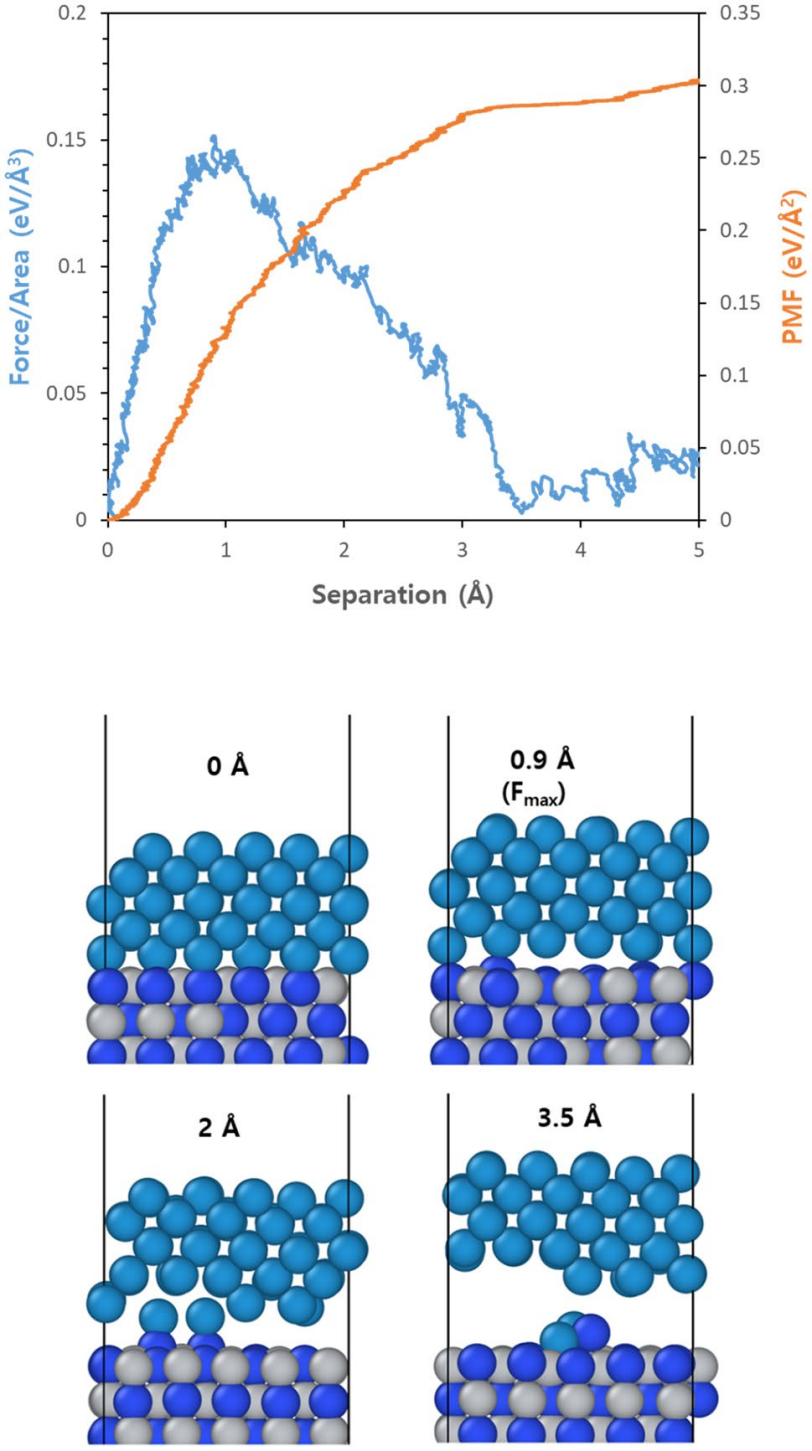
(**a**) Force (blue) and accumulated PMF (red) variations as a function of separation for W/(001)TiN structures with the unit interface when the pulling tests are applied, and (**b**) representative snapshots at four different separations lengths; W: dark cyan, Ti: gray, and N: blue.

Figure 4 demonstrates changes in adhesive properties during pulling mode on nonpolar (001)TiN structure; while showing overall similar behavior to that observed for W/(111)Ti systems as separations progress but the interface does not separate cleanly due to exposed nitrogen atoms on surfaces. Specifically, it exhibits maximum forces around 0.151 eV/Å^3^ before decreasing with some fluctuations occurring afterward. Interestingly, some nitrogen atoms on the surface, due to their strong adhesion with tungsten, move significantly away from their original positions, and thus W does not show a clean separation on the (001)TiN facet. To summarize the adhesion results on three facets, from a failure mode perspective, W/(111)Ti and W/(001)TiN exhibit adhesive failure while W/(111)N shows cohesive failure. In terms of adhesion force, W/(111)N exhibits the strongest adhesion among these three structures, followed by W/(001)TiN, and W/(111)Ti shows the weakest adhesion. This is also consistent with PMF-predicted values; however since PMF results heavily depend on atomic behaviors, careful interpretation is required. (This will be discussed further in the next section).

**Figure 5:**
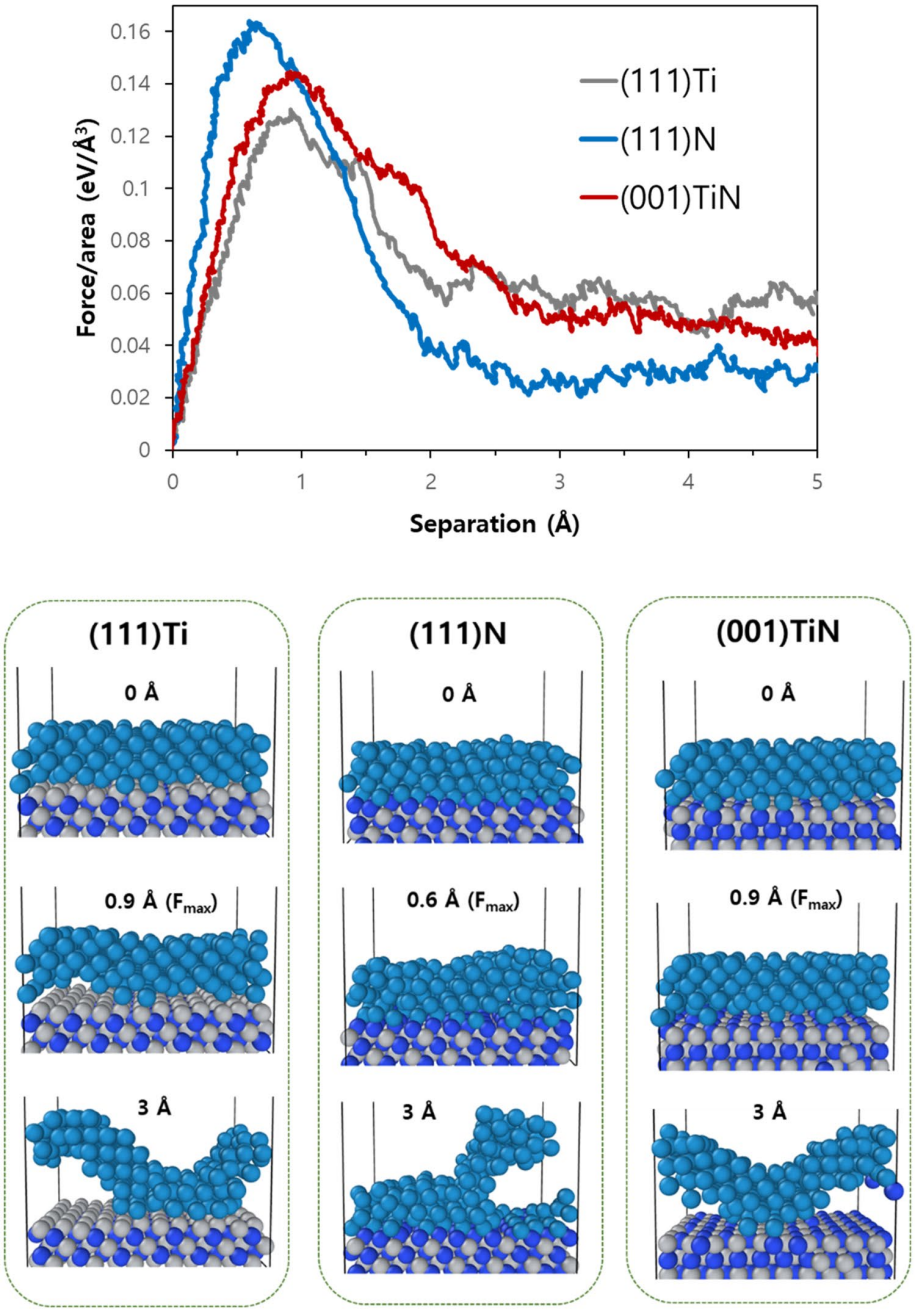
(**a**) Force variations when pulling mode is applied to three different W/TiN structures with a 2 × 2 interface. Note that a 2 × 2 interface indicates a four times larger interfacial area than the unit interface. (**b**) Representative snapshots at each structure. The number represents the separation distance after equilibrium simulation (set to 0 Å); W: dark cyan, Ti: gray, and N: blue.

To investigate whether the adhesion behavior on each facet remains consistent even as interface size increases, we performed SMD simulations with a fourfold larger interface. Figure 5a shows changes in force when W separates from TiN at each facet for this 2 × 2 interface structure. The adhesion strengths follow the same order as smaller-sized interfaces: highest for W/(111)N followed by W/(001)TiN, and then W/(111)Ti. Furthermore, comparing these values (Table 1), we can see they remain almost identical regardless of increasing interface sizes. Figure 5b shows structural changes at each facet during the pulling test in the 2 × 2 interface. It indicates adhesive failures occur for both W/(111)Ti and W/(001)TiN while cohesive failure for W/(111)N occurs, which is also identical to the failure mode obtained by unit interface structures. However detailed examination into how separation occurs reveals slight differences between two sized interfaces. For example, even though clean adhesive failure occurs like unit interface structure in some parts of the W/(111)Ti interface, some W atoms still remain on the (111)Ti surface on other interfacial regions. The interactions between W and TiN atoms constantly vary according to their local structures at each moment, and thus it is impossible to make all atoms behave identically in the large interface structure despite applying the same velocity onto all W atoms pulled outwards towards opposite direction simultaneously. As a result, since some parts still remain on the surfaces even after separations increase, pulling forces never reach zero in the 2 × 2 interface structure. Thus, accumulated PMFs (the area under force graph in Fig. 5a) is also unable to converge into specific value but rather keep increasing. In Table 1, the PMF values are arbitrarily chosen based on converged force conditions around ~ 3 Å separation at a 2 × 2 interface structure because the force values converge at that separation for all facets. This means defining adhesive energy in terms of PMF is somewhat arbitrary and its interpretation depends heavily upon dynamics involved during atomistic behavior. In particular, in the case of cohesive failure like W/(111)N, it is assumed that the interfacial structure remains intact while W itself is destroyed. At this point, the adhesive energy cannot be determined, but it is expected to be larger than the PMF value at this time. Therefore, even if the PMF value of W/(111)N is smaller than that of W/(001)TiN in the 2 × 2 interfacial structure, the W/(111)N can have a stronger adhesion in the interface. However, as stated above, the maximum forces required to separate interfaces do not vary to interface size, and thus the value is a good indicator for quantifying the adhesive strength. In summary, the failure mode is independent of interfacial size and can be consistently interpreted using the maximum necessary force required to cause such failure (whose trend and magnitude remain constant regardless of interfacial size). However, interpreting PMF values requires caution since they depend heavily on atomic dynamics involved when the failure occurs.

**Table 1:** Force and energy (PMF) per interface area on each facet.

Facet	Force/area (eV/Å^3^)		Energy/area (eV/Å^2^)	
	Unit	2 × 2	Unit (at plateau)	2 × 2 (at 3 Å)
(111)Ti	0.130	0.133	0.27 (A)	0.22 (A)
(111)N	0.164	0.163	0.32 (C)	0.22 (C)
(001)TiN	0.151	0.145	0.30 (A)	0.28 (A)

The (A) and (C) represent the adhesive and cohesive failures, respectively. Force represents the maximum force, and energy is the value when the PMF reaches the plateau and at 3 Å for the unit interface and 2 × 2 interface structures, respectively.

**Figure 6:**
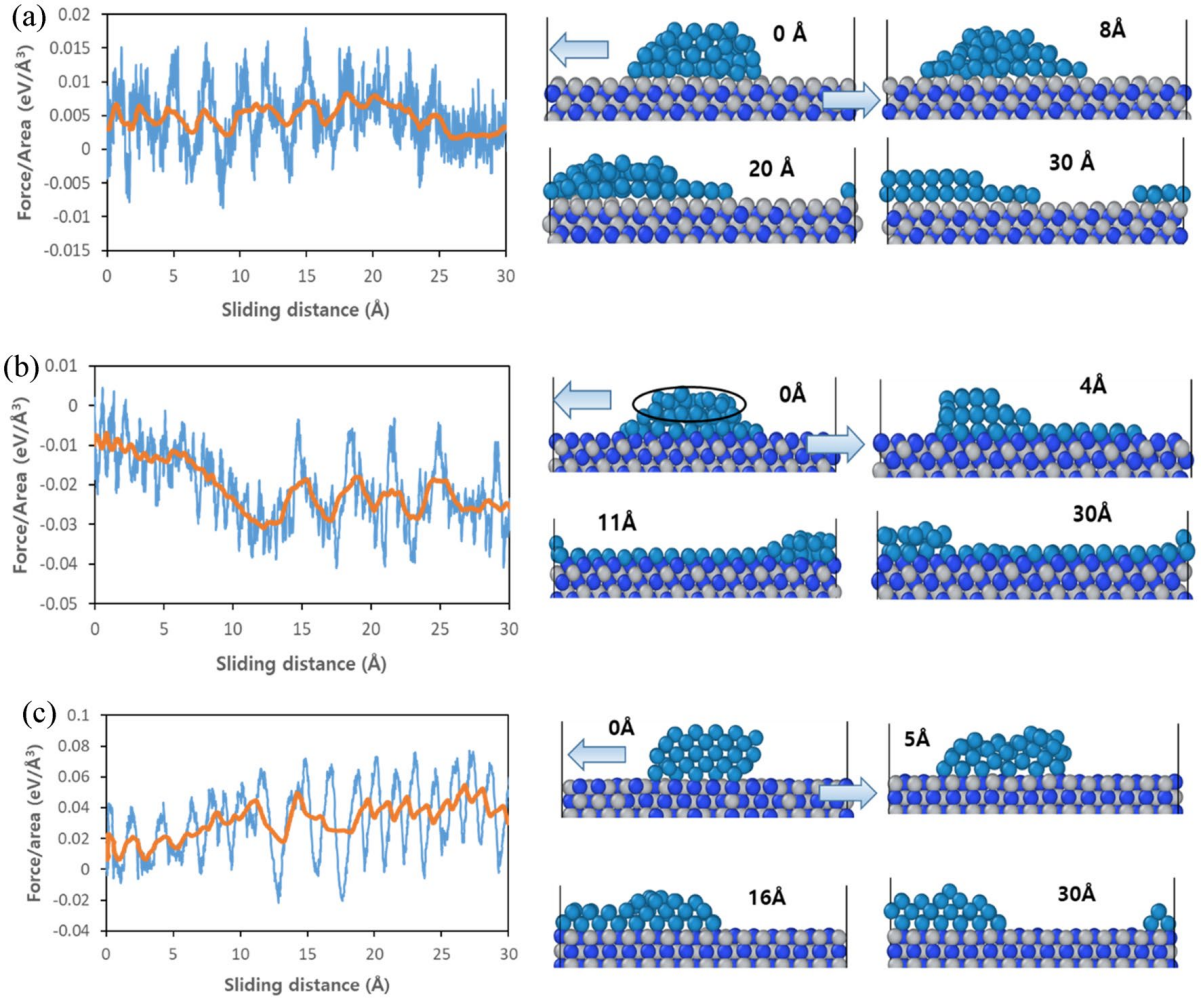
Force variations and representative snapshots when the sliding test is applied to fractional-W structure on (**a**) (111)Ti facet, (**b**) (111)N facet, and (**c**) (001)TiN facet. The red line represents the averaged force calculated by averaging the force values every 10 ps. The number in snapshots represents the sliding distance after the equilibrium process (set to 0 Å), and arrows at 0 Å show the direction of applied velocities for the sliding test; W: dark cyan, Ti: gray, and N: blue.

Figure 6 shows the force required for W to slide on each TiN facet and the resulting structural changes during the sliding test. The initial structure was set with the same W size used in unit interface structure on a three times larger TiN structure (1 × 3 interface) in the direction of sliding, and then a sliding test was conducted to clearly observe structural changes due to adhesion between W and TiN as it moves along its surface. For W/(111)Ti in Fig. 6a, during the initial sliding phase, the force value shows fluctuations between − 0.005 eV/Å^3^ and 0.015 eV/Å^3^, which is one order smaller than the maximum force of the pulling test. Moreover, as the sliding test continued (i.e., with an increase in sliding distance), these fluctuations decreased. The positive force value indicates that W is moving in the opposite direction of TiN (i.e., the direction of the sliding test), meaning that the distance between W and TiN is increasing. On the other hand, negative force means that overall W atoms move in a similar direction as TiN (even if they move locally in a different direction), meaning that they are getting closer together. To clarify why there are negative forces, snapshots at some sliding distances are shown. When W slides on (111)Ti surface, it cannot maintain its initial shape, and some parts spread towards the empty surface side with exposed Ti atoms while moving. In this case, some of the W atoms that spread out on the surface exhibit a negative force, giving the effect of moving in the same direction as TiN. However, overall, W tends to move in the opposite direction of TiN, thus showing a positive force. On the other hand, considering the small value of the force, it is expected that W can easily move on the (111)Ti surface.

On the other hand, as shown in Fig. 6b, when W undergoes a sliding test on (111)N facet, the force value gradually increases in the negative range as the sliding distance increases before fluctuating within this region after passing through 11 Å of sliding distance. The magnitude of negative forces initially increases and reaches its maximum value greater than − 0.03 eV/Å^3^ which is more than six times larger compared to those obtained during testing over W/(111)Ti structure. Snapshots show how W atoms present at the interface combine with surface N atoms and move together in the same direction while most of the W atoms on upper layers (marked by ellipses within the figure) speedily move towards opposite directions relative to TiN, spreading in the bare surface, and leads to the generation of negative forces. As the test continues further all W atoms cover the entire (111)N facet and those directly bonded with surface N atoms remain moving along TiN, while some other W atoms located on upper layers continue to move oppositely to the nitride surface causing fluctuations in negative force values. Hence, it can be concluded that due to strong adhesion at the interface for W/(111)N, it would be difficult for W atoms located on the interface to move parallel to the surface against N atoms.

For W/(001)TiN structure (Fig. 6c) where half of the surface is exposed with N atoms, unlike in the case of W/(111)N, force values according to sliding distance mostly show positive values indicating that W moves in a different direction from TiN. However, it requires very large forces exceeding 0.06 eV/Å^3^ at a maximum value that is about four times larger than the maximum force (0.015 eV/Å^3^) observed during testing over (111)Ti facets. Therefore, it can be expected that sliding will be relatively difficult on (001)TiN compared to (111)Ti facet. In summary, for both (111)Ti and (001)TiN facets, although some atoms tend to spread towards the surface side during sliding tests, overall W moves towards opposite directions relative to TiN generating positive forces. However, (111)N surface has strong bonding between its surface N atoms and tungsten causing the W atoms located on upper layers rapidly spread on the nitride surface, while interfacial W atoms directly bonded with the surface N atoms move alongside them thereby generating large negative forces. Meanwhile, sliding tests were also conducted on the structures where W completely covers (Full-W structure) each surface of TiN metal (Fig. S3 in SI). All structures show positive forces and this is because, unlike the fractional-W structure in Fig. 6, there are no bare surfaces available on TiN, so most parts of W atoms tend to move along the sliding test direction. The W sliding is found to be more difficult on the (001)TiN facet than on the (111)Ti facet as observed in the fractional-W structure. However, for the (111)N facet, the sliding force is found to be larger than that of the (111)Ti facet, but smaller than that of the (001)TiN facet. This is because tungsten atoms on and above the interface do not move in unison for W/(111)N structure. The W atoms at the interface tend to move towards the TiN direction due to strong bonding with surface N atoms, while those above tend to move oppositely causing resultant forces between them leading toward small values. Therefore, it can be expected that as seen during pulling tests where cohesive failure was observed due to strong interfacial adhesion for W/(111)N structure, similarly during sliding tests also tungsten located on or near the interface would have difficulty in sliding compared to those above.

## Conclusions

The failure mechanism of the device caused by the atomic migration phenomena occurring at the interface of a metal interconnect is closely related to the interfacial adhesion, and thus understanding the adhesion characteristics of the interface is crucial for ensuring long-term reliability. There are various experimental methods for measuring adhesion, but it is difficult to provide consistent interpretations due to differences in the measurable materials, measurement methods, and fundamental theories. As an alternative to this, our study proposes a method for theoretically understanding the adhesive properties through steered molecular dynamics simulations based on machine learning potential. We investigated the adhesive behaviors of tungsten deposited on barrier metal TiN, which are representative metal interconnect structures. When performing a pulling test on the two materials, in the case of tungsten deposited on (111)Ti and (001)TiN facets, it shows adhesive failure. However, when it is deposited on (111)N facet, the internal bonding of tungsten is broken due to strong adhesion at the interface and shows cohesive failure. We have demonstrated that adhesion strength can be defined as the maximum force required to separate the interface. It was found that (111)N facet shows the strongest adhesion and (111)Ti shows the weakest when tungsten is deposited on it. In sliding tests, where two materials move on each other on the interface, a force one order of magnitude smaller than in pulling tests was measured. As seen in the pulling results, tungsten requires the least force to slide on the (111)Ti facet. It can be expected that for the (111)N facet, due to the strong bonding between tungsten and nitrogen, tungsten rapidly spreads towards the nitride surface and is hard to slide on the interface. Finally, this modeling method, which combines steered molecular dynamics simulations and machine learning potential, is expected to contribute to enhancing the understanding of the adhesive properties of various metal interconnect devices as it can be easily applied to other metal interfaces.

### Supplementary Information

Supplementary Information.

## Data Availability

The database used in this study can be requested via the corresponding author upon reasonable request.
