# Supplementary material for: Atomistic insights into adhesion characteristics of tungsten on titanium nitride using steered molecular dynamics with machine learning interatomic potential
Source: Sci Rep. 2023 Oct 10;13:17145. doi: 10.1038/s41598-023-44265-6 (PMC10564797; doi:10.1038/s41598-023-44265-6)

**Supporting Information**

**Atomistic Insights into Adhesion Characteristics of Tungsten on Titanium Nitride using Steered Molecular Dynamics with Machine Learning Interatomic Potential**

Eunseog Cho^1,^*, Won-Joon Son^1,^*, Eunae Cho^1^, Inkook Jang^1^, Dae Sin Kim^1,^ and Kyoungmin Min^2,^*

^1^CSE team, Samsung Electronics, 1 Samsungjeonja-ro, Hwaseong-si, Gyeonggi-do, 18448, Republic of Korea

^2^School of Mechanical Engineering, Soongsil University, 369 Sangdo-ro, Dongjak-gu, Seoul 06978, Republic of Korea

**Calculation Details**

**I-1. TiN slab structures and amorphous-like W**

The (001)TiN facet is a nonpolar one where Ti and N coexist in the same layer, allowing for the creation of slab structures with the same composition ratio as bulk stoichiometry. However, for (111) polar facet, since Ti and N alternate to form layers, creating the slab structure with the same stoichiometry as the bulk structure which results in a dipole moment existing perpendicular to the layers. According to the Taker theory, if a dipole moment exists in the vertical direction of a layer, the surface energy becomes infinite. Therefore, to prevent unrealistic surface structures from forming, symmetric slab structures with identical atoms on both top and bottom surfaces must be created. For example, for Ti-rich (111) facets ((111)Ti), slab structures with only Ti atoms present on both top and bottom surfaces should be created; conversely, for N-rich (111) ((111)N), symmetric slab structure with only N atoms present on both sides should be made. In this modeling, we constructed 5-7 layer slabs depending upon each facet orientation while creating an amorphous-like W structure using the simulated annealing method. Specifically, bulk W structure was melted at 3000K temperature through MD simulation for 500 ps followed by gradual cooling down to room temperature of 300K over another period of MD simulation lasting an additional 500 ps using a quenching method resulting in the intermediate amorphous-like state between initial crystal and melt states as shown in Figure S1 where MTP potentials were developed based upon constructing various types of W structural models including bulk, molten, and quenched ones.

**Figure S1**. The RDF distribution of three different tungsten structures; bulk W, molten W at 3000K, and quenched (amorphous-like) W at 300K.


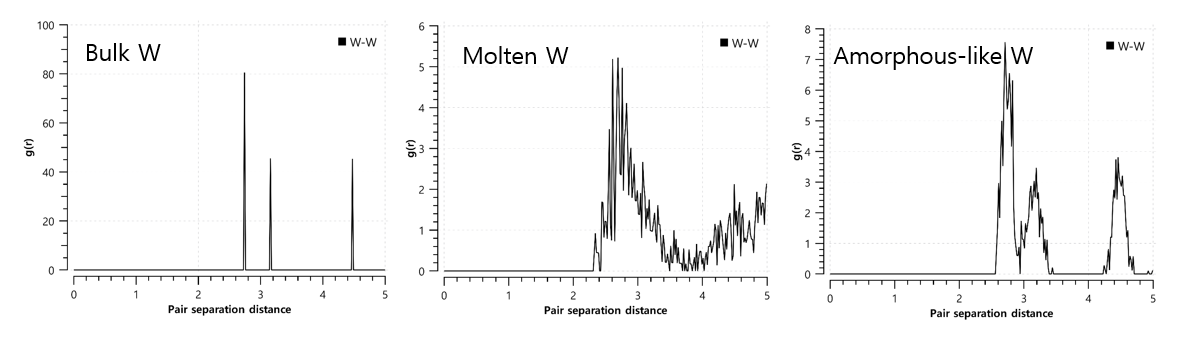


[1] Tasker, P. W. The Stability of Ionic Crystal Surfaces. *J. Phys. C: Solid State Phys.* **1979**, *12*, 4977.

**II. DFT calculation details**

The first-principles calculations were performed by the pseudopotential plane wave method, using the Vienna ab initio Simulation Package (VASP). We adopted the generalized gradient approximation (GGA) implemented by Perdew, Burke, and Ernzerhof (PBE) for the exchange-correlation energy functional with the D2 correction of Grimme to treat the van der Waals (vdW) interactions between W and TiN. The energy cutoff was selected to be 400 eV, and the atomic positions for bulk and slab structures were fully relaxed until the ionic force on each atom was below 0.02 eV/Å. The energy convergence criteria are set to be 10^-5^ eV. A dipole correction in the z-direction (perpendicular to the surface) is additionally used for an energy calculation of W/TiN film structures. A vacuum region of more than 20 Å is also selected along the z-direction to avoid spurious effects stemming from the periodic boundary condition. Ab initio MD simulations were performed at 300K using a Nose-Hoover thermostat.

**Figure S2.** Force and accumulated PMF variations per interfacial area as a function of the separation when W is pulled from (111)Ti, (111)Ni, and (001)TiN facets in 2x2 interface structures; blue line: Force, red line: PMF.


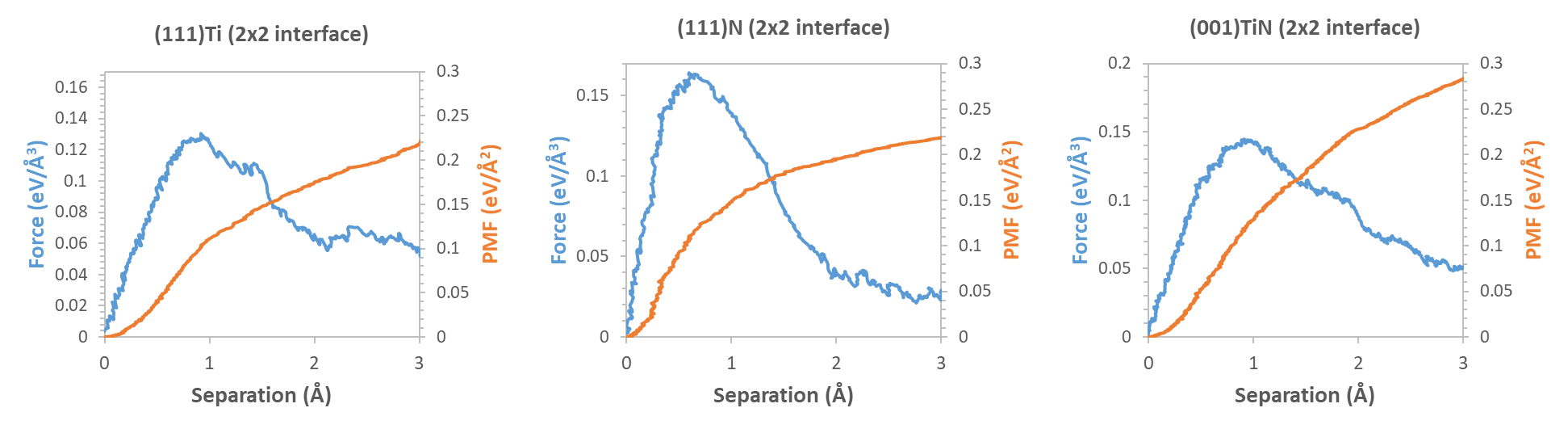


**Figure S3** (a) Force variations when sliding testing mode is applied to three different TiN/W structures. The red line represents the averaged force calculated by averaging the force values every 10 ps. (b) Representative snapshots at each structure. The number represents the sliding distance after the equilibrium process (set to 0 Å); W: dark cyan, Ti: grey, and N: blue.


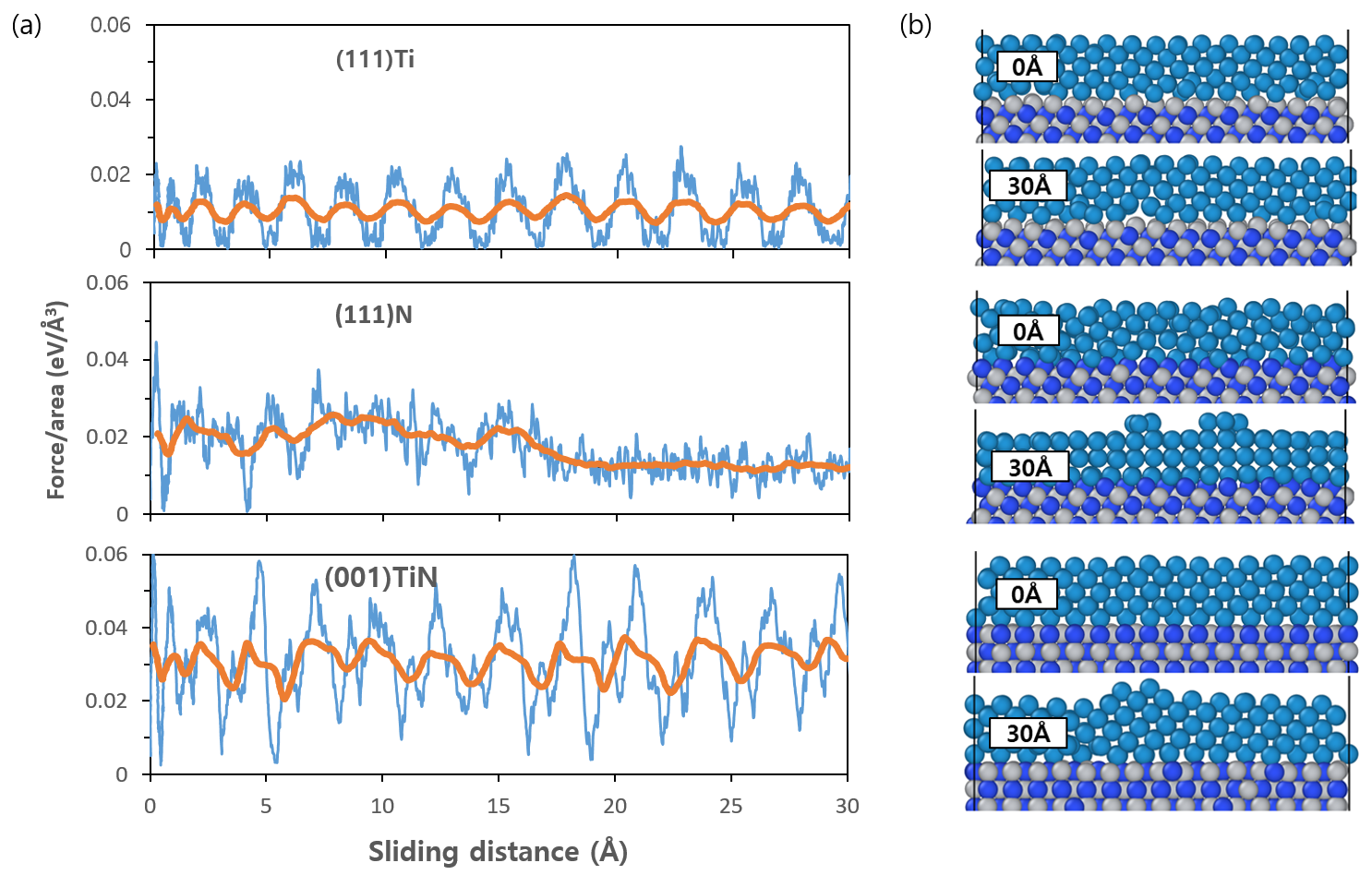

Supplement: Supplementary file 1 — Supplementary Information. [file 41598_2023_44265_MOESM1_ESM.docx]
